# Resistance Training Causes the Stretch-Induced Force Deficit—A Randomized Cross-Over Study

**DOI:** 10.3390/sports12060145

**Published:** 2024-05-27

**Authors:** Konstantin Warneke, Katharina Turau, Lars Hubertus Lohmann, Martin Hillebrecht, David G. Behm, Andreas Konrad, Tobias Schmidt

**Affiliations:** 1 Institute of Human Movement Science and Exercise, University of Graz, 8010 Graz, Austria; andreas.konrad@uni-graz.at; 2 Institute of Sport Science, Alpen-Adria University Klagenfurt, 9020 Klagenfurt am Wörthersee, Austria; 3 Institute of Interdisciplinary Exercise Science and Sports Medicine, MSH Medical School Hamburg, 20457 Hamburg, Germany; katharina.turau@medicalschool-hamburg.de (K.T.); tobias.schmidt@medicalschool-hamburg.de (T.S.); 4 University Sport Center, Carl von Ossietzky University Oldenburg, 26129 Oldenburg, Germany; lars.hubertus.lohmann@uni-jena.de (L.H.L.); hillebre@uni-oldenburg.de (M.H.); 5 Institute of Human Movement Science and Exercise Physiology, Friedrich-Schiller University Jena, 07743 Jena, Germany; 6 School of Human Kinetics and Recreation, Memorial University of Newfoundland, P.O. Box 4200, St. John’s, NL A1C 5S7, Canada; dbehm@mun.ca

**Keywords:** acute stretching, range of motion, calf muscle, maximal strength, dose–response relationship

## Abstract

Purpose: Stretch-induced force deficit suggests an acute stretch-specific strength capacity loss, which is commonly attributed to EMG reductions. Since those deficits could also be attributed to general fatigue induced by overloading the muscle, this study aimed to compare stretching with an exhausting calf raise programme to compare strength and stretching responses. Method: This study included 16 participants with different, high-duration calf muscle stretching effects (10, 20, 30 min of stretching) with resistance training (RT) (3 × 12 repetitions) performed until muscle failure, by using a cross-over study design with pre-post comparisons. Strength was tested via isometric plantar flexor diagnostics, while flexibility was assessed using the knee-to-wall test (KtW) and an isolated goniometer test. Results: Using a three-way ANOVA, RT strength decreases were greater compared to 10 and 20 min of stretching (*p* = 0.01–0.02), but similar to those of 30 min of stretching. ROM in the KtW showed no specific stretch-induced increases, while only the stretching conditions enhanced isolated tested ROM (*p* < 0.001–0.008). No RT-related isolated ROM increases were observed. Conclusions: The results showed both interventions had similar effects on strength and ROM in the calf muscles. More holistic explanatory approaches such as fatigue and warm-up are discussed in the manuscript and call for further research.

## 1. Introduction

Although static stretching acutely enhances flexibility [1,2], many studies have described subsequent performance impairments with maximal- and speed strength-related parameters [3,4,5]. These stretch-induced force deficits typically occur in response to stretching interventions exceeding 60 s per muscle group [3,5,6]. For example, Kokkonen et al. [7] showed that 5 stretches, each performed 6 times for 15 s, reduced strength capacity by about 8%. While Leone et al. [8] showed 2 × 30 s stretching significantly impaired upper body strength performance, Siatras et al. [9] showed 60 s of static quadriceps stretching to be sufficient to reduce maximal strength production significantly. Increasing the stretching time, Marchetti et al. [10] showed detrimental effects of 23.6% caused by 3 × 40 s high-intensity static stretching at 85% of the point of discomfort (POD). Bacurau et al. [11] reported a maximal strength loss from 213.2 ± 36.1 kg to 184.6 ± 28.9 kg in response to 20 min of static stretching. To date, the longest stretching times were assessed by Warneke et al. [12] who measured an acute maximum strength loss of 16.2% after performing one bout of 1 h continuous static stretching of the plantar flexors. 

Performance decreases were extensively discussed in the Behm and colleague reviews [5,13], suggesting stretching-induced changes in neuromuscular activation patterns [14], motoneuron excitability including central and peripheral reflex mechanisms [15,16] as well as reduced strength of persistent inward currents (PICs) [17] at the motoneuron dendrites [18], all of which could contribute to reduced force production. Furthermore, possible morphological changes such as reduced muscle-tendon unit stiffness [19] or thixotropic effects (reduced visco-elasticity) [20] might also affect force production since deficits in the ability to store and transfer energy [21] would adversely affect the stretch-shortening cycle.

These force and performance deficits have been ubiquitously attributed to prolonged static stretching [5,6,22]. As it is primarily prolonged duration stretching which induces the impairments, the deficits might be associated with general fatiguing effects, rather than the stretching action itself. Innumerable studies report fatigue-induced deficits following resistance training [23,24,25]. In the direct comparison, Warneke et al. [12] showed similar decreases in maximum force production in response to either 5 sets of 12 seated calf raise repetitions or 1 h stretching. While full ROM resistance training was described as a type of dynamic stretching [26] that is known to acutely enhance ROM as well, Warneke et al. [12] hypothesized simple warm-up effects to be more likely than specific RT effects. Even though no final explanation can be provided, there is sufficient evidence that full ROM RT can meaningfully increase ROM (30). Thus, if both RT and stretching can increase ROM and induce performance impairments, are there commonalities in their responses? This study explored (1) if there was a dose–response relationship in force production ability using different long-duration stretching protocols and (2) the comparison to an exhausting resistance training routine to induce muscle fatigue and compare these effects with those of the stretch-induced force deficit to determine if there are shared underlying responses for fatigue-induced decrements and ROM improvements.

## 2. Methods

### 2.1. Experimental Design

**Figure 1:**
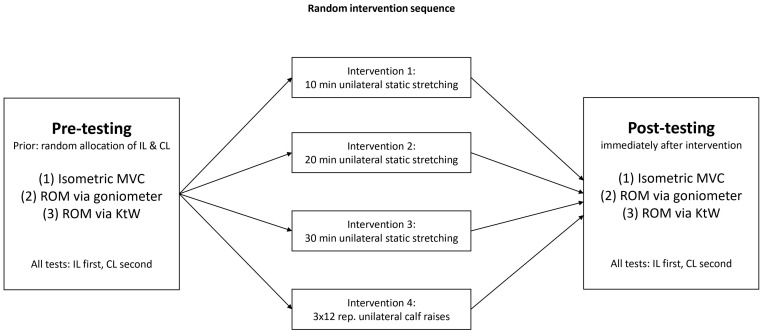
Illustrates the study procedure. CL = control leg, IL = intervened leg, KtW = knee to wall, MVC = maximal voluntary contraction, ROM = range of motion.

The interventions were conducted on four, non-consecutive days. In the stretching conditions, participants performed either 10, 20, or 30 min of unilateral plantar flexors static stretching, while the strength training condition consisted of 3 sets of 12 repetitions of unilateral calf raises using a Smith machine. The intervention was performed using the dominant leg determined by which leg would predominately kick a ball, while the non-dominant leg served as the intra-individual control. The acute effects of the different interventions on plantar flexors maximal strength and dorsiflexion ROM with extended and flexed knee joints were investigated (see Figure 1).

### 2.2. Participants

Sample size estimation was performed using G*Power sample size estimations via F-test ANOVA with repeated measures (two measurements and four conditions) within-between interactions, estimated by applying f = 0.4, alpha error = 0.05, and power = 0.80 [12] resulting in a required sample size of n = 16.

Consequently, 16 recreationally active participants (m: n = 9, age: 25.57 ± 4.13 years, height: 181.36 ± 9.06 cm, weight: 92.55 ± 17.92 kg, f: n = 7, age: 25.57 ± 4.13 years, height: 181.36 ± 9.06 cm, weight: 92.55 ± 17.92 kg) were recruited from the university sports campus. All participants were randomly allocated to four exercise conditions via the Excel randomization function (RANDbetween). This resulted in a total of 64 pre- to post-test comparisons. Participants were considered recreationally active if they regularly performed resistance training at least twice a week and confirmed that they were injury-free for the last six months. Therefore, only participants with experience in resistance and /or flexibility training and participation in studies performing strength and ROM measurements were eligible for participation. Participants who reported injury that caused immobilization within the last six months, untrained or elite sport athletes as well as those without experience in isometric strength and ROM testing protocols were not considered for participation. All participants provided written informed consent. The study was performed in accordance with the Helsinki Declaration and approved by the local ethical review board (Carl von Ossietzky University Oldenburg, No.121-2021). 

#### Testing Procedure

Prior to testing, a warm-up routine was performed using five minutes of ergometer cycling at 60 rpm. Afterwards, participants performed the maximal plantar flexors strength test, followed by the dorsiflexion (DF) ROM test with the extended knee joint and the knee-to-wall test (KtW) as a functional flexibility test [12]. 

### 2.3. Maximal Voluntary Isometric Contraction (MVIC) Strength

MVIC strength was assessed using single-leg testing with an extended knee joint in a horizontal leg press. A 50 × 50 cm force plate, with a range of ±5000 N and a 13-bit analogue-to-digital converter and a collection frequency of 1000 Hz was attached to the footpad to record the maximal strength. MVIC strength measurement was performed by placing the forefoot on the lower edge of the attached force plate, using an ankle joint angle of 90°. To measure plantar flexor MVIC strength, the force plate was fixed in a standardized position to form an immovable resistance. The participants were instructed to perform a plantar flexion against the force plate as hard as possible which was held for three seconds. This process was repeated until no improvements in the recorded MVIC strength values were achieved with a minimum of four trials. To avoid fatigue, a recovery of 1 min between trials was provided. 

All tests were performed unilaterally.

### 2.4. Dorsiflexion (DF) ROM with Extended Knee Joint

DF ROM with an extended knee joint was evaluated using a goniometer attached to a stretching device, which was also used to induce a standardized constant angle of stretching. This procedure was previously described by [12]. A high test-retest reliability with an ICC = 0.995 was previously reported. Participants positioned their foot on a chair with the knee joint held in an extended position, while the ankle was fixed in the stretching device. The investigator pushed the foot into the maximal dorsiflexed position until the participant reported reaching the point of maximal tolerable discomfort. The ankle angle [°] was then read from the attached goniometer. This procedure was performed twice, the maximum value was used for further calculation.

### 2.5. Dorsiflexion ROM Measurement with Flexed Knee Joint Using the Knee to Wall Test (KtW)

**Figure 2:**
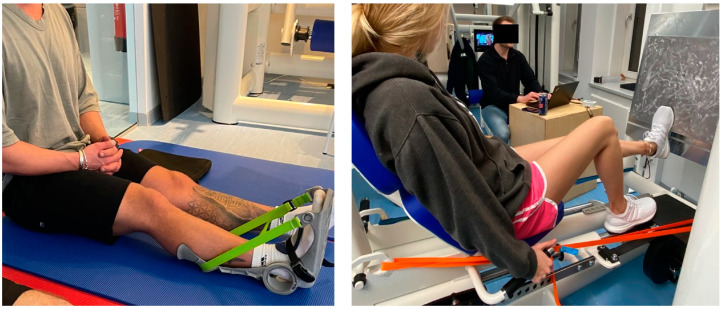
Illustrates the flexibility testing as well as the isometric maximal strength test.

The KtW measurement was performed to assess DF ROM with the knee joint flexed, which has been frequently performed when evaluating functional ankle joint ROM [27]. The participants were instructed to push their knee towards the wall as far as possible while the foot was positioned on a measurement tape (Figure 2). When the heel lifted, the measurement was recorded and the corresponding distance to the wall (in cm) was documented. The mean of two trials was used for analysis. In similar investigations, authors reported high reliability when using the knee-to-wall test with ICC = 0.99 [12].

#### Intervention

Stretching or strength training was performed unilaterally, while the contralateral non-intervened leg was used as intra-individual passive control, as no intervention was applied. Standardized stretching was performed with the calf muscle stretching device used for ROM measurement with the extended knee joint. The participants were instructed to wear the stretching device on their dominant leg for the prescribed duration (10, 20, or 30 min). When starting the stretch, the participants were instructed to report the stretching pain via a visual analogue scale (7 out of 10). Resistance training was performed with (extended knee joint) standing calf raises using a Smith machine. The knee joint was held in an extended position, which was checked by two investigators during data collection.

The exercise was conducted with 3 sets of 12 repetitions with a weight that could be moved over the full ROM for 10 to 12 times. If less than 10 repetitions were possible, 5 kg were removed. The movement was performed over the full ROM.

## 3. Data Analysis

The analysis was performed with SPSS 28.0. Normal distribution was checked via the Kolmogorov Smirnov test (n > 30), while reliability was reported using the interclass correlation coefficient (ICC) and the coefficient of variability (CV). A three-way ANOVA (4 conditions × 2 tests × 2 legs) was conducted on maximal strength and flexibility data. To receive further insights, a series of 2 × 2 ANOVAs (Leg × Time) for each condition as well as ANOVAs (Condition × Time) separated for legs were performed. Effect sizes were presented as Eta squares (η^2^) and categorized as small effect η^2^ < 0.06, medium effect η^2^ = 0.06–0.14, large effect η^2^ > 0.14. The between-condition differences revealed in the post-hoc test for the condition × Time analysis was described via Cohen’s d, classified as small: 0-0.5, moderate: 0.5–0.8, large: >0.8 [28]. The level of significance was set at *p* < 0.05. 

## 4. Results

No significant pre-test differences were detected (*p* = 0.893–0.999). With ICC = 0.996, CV = 1.81% in the MVIC strength testing and with an ICC = 0.977–0.982, CV = 2.6–3.5%, the reliability of the ROM testing procedure was classified as excellent [29]. Table 1 and Table 2 provide descriptive statistics and statistical key values, respectively.

### 4.1. Effects of Stretching Intervention and Strength Training on MVC

A large magnitude Time effect (η^2^ = 0.81, *p* < 0.001) indicated pre-post changes, while effects for Leg and Condition did not reach the level of significance (*p* = 0.42–0.85). The 3-way ANOVA revealed significant interactions for Leg × Time (η^2^ = 0.62, *p* < 0.001) and the three-factor calculation Leg × Time × Condition (η^2^ = 0.57, *p* = 0.02).

#### 4.1.1. 2 × 2 ANOVA (Leg × TIME)

Ten (10) and 20 min of stretching showed no significant decrease in MVC (η^2^ = 0.04–0.24, *p* = 0.07–0.83) without a difference between the IL and CL (η^2^ = 0.001–0.02, *p* = 0.10–0.91), while 30 min of stretching induced a significant decrease in MVIC force (η^2^ = 0.52, *p* = 0.003), however, without a difference between the legs. Only the resistance training conditions showed a high magnitude decrease (η^2^ = 0.43, *p* = 0.008) with an interaction (η^2^ = 0.62, *p* < 0.001), indicating a larger decrease in IL compared to CL. 

#### 4.1.2. 2 × 2 ANOVA (Condition × Time)

**Figure 3:**
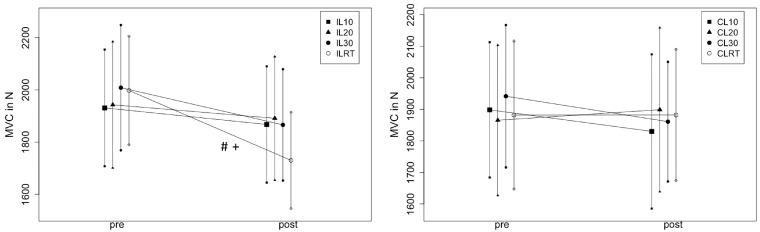
Illustrates the maximal strength effects in the intervened (IL) and control (CL) leg. # = significant difference compared to IL10, + = significant difference compared to IL20, CL = control leg, IL = intervened leg, MVC = maximal voluntary contraction, RT = resistance training.

In the IL, there was a large magnitude MVIC decrease from pre- to post (Time: η^2^ = 0.36, *p* < 0.001) with an interaction (η^2^ = 0.20, *p* = 0.009) that indicated a condition-dependent MVIC decrease (Figure 3). The post-hoc testing showed the strongest decrease for the resistance training condition with significant differences to the 20 min (*p* = 0.01, d = 0.72) and 10 min (*p* = 0.02, d = 0.68) stretching conditions, without a difference to the 30 min (*p* > 0.05. With η^2^ = 0.04, *p* = 0.15 and η^2^ = 0.10, *p* = 0.13. There was neither a Time- nor a Time × Condition Interaction in any condition reported for the CL.

### 4.2. Effects on ROM

ROM testing with extended knee joint using the goniometer to test isolated plantar flexor ROM.

The three-way ANOVA revealed a significant Time effect (η^2^ = 0.85, *p* < 0.001) without Leg and Condition reaching the level of significance (*p* = 0.21–0.70). With η^2^ = 0.56, *p* = 0.001 and η^2^ =0.62, *p* = 0.011, the two-factor interactions Leg × Time and Time × Condition as well as the three factor Leg × Time × Condition (η^2^ = 0.76, *p* < 0.001) showed large magnitude effects. 

#### 4.2.1. 2 ×2 ANOVA (Leg × Time)

All stretching conditions induced a large ROM increase (η^2^ = 0.55–0.67, *p* < 0.001) showing the intervened leg with higher increases (Interaction: η^2^ = 0.43–0.77, *p* < 0.001–0.008). There were near significant ROM increases in the resistance training condition, (η^2^ = 0.26, *p* = 0.06) with no significant Leg × Time interaction (η^2^ = 0.25, *p* = 0.06) was observed.

#### 4.2.2. 2 ×2 ANOVA (Condition × Time)

**Figure 4:**
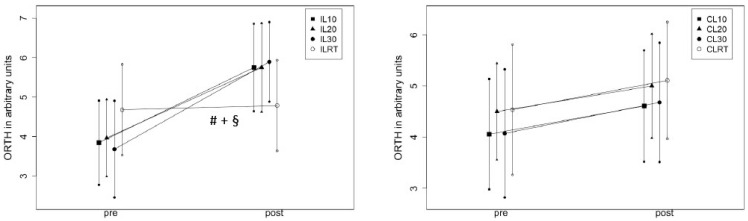
Illustrates the ROM changes with the orthotic device in the intervened (IL) and the control leg (CL). # = significant difference compared to IL10, + = significant difference compared to IL20, § = significant difference compared to IL 30, CL = control leg, IL = intervened leg, RT = resistance training, ORTH = ROM measuring via the goniometer of the orthosis.

In the IL there was a large ROM increase (Time: η^2^ = 0.67, *p* < 0.001) with an interaction effect (η^2^ = 0.38, *p* < 0.001) showing superior effects of stretching compared to the RT conditions (*p* < 0.001–0.001, d = 0.92–1.16) without differences between the stretching conditions (*p* = 0. 735–0.993). In CL, there was a flexibility increase (η^2^ = 0.37, *p* < 0.001) without a difference between conditions (η^2^ = 0.003, *p* = 0.98). Changes are graphically illustrated in Figure 4.

ROM testing with flexed knee joint using the knee-to-wall test.

In the 3-way ANOVA, only the Time effect reached the level of significance (η^2^ = 0.56, *p* = 0.001), while all interactions remained non-significant (η^2^ = 0.10–0.27, *p* = 0.23–0.82). 

#### 4.2.3. 2 × 2 ANOVA (Leg × Time)

Accordingly, all IL (except the 20 min stretching condition) showed ROM increases compared to the CL (η^2^ = 0.35–0.62, *p* < 0.001–0.02), without showing interaction effects (η^2^ = 0.02–0.12, *p* = 0.20–0.92). 

#### 4.2.4. 2 × 2 ANOVA (Condition × Time)

In both legs, there was a ROM increase (IL: η^2^ = 0.21, *p* < 0.001, CL: η^2^ = 0.17, *p* = 0.002) without an interaction (*p* = 0.59–0.99).

## 5. Discussion

The present study compared the acute effects of 10, 20, and 30 min of static stretching with those of a typical resistance training stimulus on the plantar flexors MVIC strength and ROM. In accordance with current literature, stretching enhanced ROM and caused a stretch-induced force deficit ranging between 2.7–7.1%, while about a 13% impairment was reported for RT. However, 10 and 20 min of stretching did not significantly reduce MVIC strength capacity, while 30 min of stretching and RT impaired maximal strength capacity without a significant difference between the conditions.

While all conditions increased KtW measure (DF ROM) with similar magnitude in both, IL and CL (except for 20 min stretching), only stretching increased ROM in the specific stretching device. ROM testing with an extended knee joint showed a peak increase in the 30 min condition. In contrast to KtW, RT did not improve flexibility with the specific stretching device.

### Stretch-Induced Force Deficit

In accordance with previous literature [3,4] the present study revealed a stretch-induced force deficit ranging from 2–7%. However, with more than a 13% impairment, RT nearly doubled the deficit. In previous research, Warneke et al. [12] showed similar acute plantar flexor strength decreases of −15.5–−16 ± 2.0% in response to 5 × 12 repetition 45° leg press calf raises in comparison to one hour of stretching. Therefore, the so-called “stretch-induced force deficits” seem not stretch-specific, as they can also be induced via RT. However, in their systematic review, Alizadeh et al. [26] suggested full ROM RT could be considered a type of (loaded) dynamic stretching, as a stretching stimulus occurs. Accordingly, “loaded dynamic” stretching” could contribute to the fatigue associated with RT-induced force deficit”.

Hence, questions arise about the shared potential underlying mechanisms that could cause this force reduction. One hypothesis that remained unchecked with stretching is general fatigue effects, which was earlier suggested by Trajano et al. [15]. With loaded dynamic stretching (RT), a higher stimulus could be induced by enhancing the training load. Although stretching intensities may not be able to reach the stresses associated with higher load RT, prolonged stretching durations may induce fatigue through tissue structural damage. Accordingly, Behm and St-Pierre, [30] reported that longer contraction durations elicited greater fatigue-induced decrements in muscle contractile properties, which can also be observed in the present results. Smith et al. (1993) performed 17 static or ballistic stretching exercises for 3 × 60 s each and reported a significant creatine-kinase increase (CK), indicating micro-traumatization of the contractile tissue [31]. However, even though significant, Wohlann et al. [32] critically discussed the magnitude of such CK increases as clinically irrelevant. Another possibility from a rat study suggests that prolonged static stretching can restrict muscle blood flow [33], which could adversely impact metabolic activity [34] and cause fatigue [35] and thus impair force [36]. However, as there are no studies investigating structural stretching effects, this explanation remains speculative.

Further investigations regarding underlying mechanisms might be necessary. Literature suggests attributing the stretch-induced force deficit to reduced electromyography (EMG) activity immediately after stretching, decreased sympathetic nervous system activation or stretch-induced delayed reflex responses (H-reflex) [13]. However, while different reflex mechanisms were postulated to be unlikely due to their short duration post-stretch [5], diminished EMG activity is underlined by more evidence [13]. However, the questions arise to what extent reduced EMG activity can be considered stretch-specific or a greater contribution from duration-induced fatigue. Unfortunately, studies comparing the effects of stretching with alternative training routines are scarce, while completely lacking EMG evaluation. Therefore, further research is necessary to confirm or falsify stretch-specific EMG reductions as the major contributor to the stretch-induced force deficit. 

Even though evidence exists for reduced force production, further research should assess the specificity of neuromuscular and structural effects of stretching interventions, to justify the terminology of “stretch-induced force deficit” and to explore whether or not these effects can be attributed to general fatiguing effects.

## 6. Range of Motion

Stretching acutely enhanced ROM. However, several other interventions such as jogging, cycling, hot water baths, or vibration training have shown similar ROM improvements [37], interestingly, without a significant difference between interventions. Accordingly, since there was no significant difference between stretching and the other dynamic interventions in terms of muscle stiffness/passive peak torque, other possible mechanisms may be attributed to enhanced synovial fluid flow reducing joint friction [38] and thixotropic effects [20]. 

Accordingly, the present data showed almost all conditions and both legs increased ROM in the KtW, indicating no specific intervention effects. Therefore, global and/or contralateral stretching effects [39] could possibly be attributed to warm-up effects which would cause ROM increases in the contralateral limb as well. However, since only one study previously reported temperature increases in response to stretching, further research is warranted to check this hypothesis. Also, the frequently proposed central adaptations, including enhanced passive peak torque in the end ROM might be attributed to general warm-up routines. However, further research is necessary on this topic.

In contrast, isolated ROM testing via the goniometer of the orthosis exclusively showed increased flexibility in response to the stretching interventions. These results are in contrast to previous flexibility enhancements after 5 × 12 repetitions of 45° leg press raises. While, on the one hand, these results could indicate a training–testing specificity, on the other hand, it could be speculated that the flexed hip joint in the 45° leg press could induce a higher stretching degree to the ischiocrural muscle and/or innervating nerve which induced a stretch [12], while in the present study, the standing calf raise variant targeted only the calf muscle and induced a smaller stimulus. This hypothesis would highlight potential nerve-directed aspects [40], which have to be further investigated in future studies to clarify specific aspects of stretch-induced acute ROM effects. 

## 7. Limitations

The study did not include a passive control condition which would be needed to account for contralateral effects. A further limitation can be seen in the lack of providing values from a habituation session. However, participants were familiar with the testing procedure, which is highlighted by the high ICC reliability ratios (>0.9, [29]) indicating a stable testing procedure. Underlying mechanisms were not examined in this study. Controlling muscle and body temperature as well as muscle and tendon stiffness, blood flow and EMG activity could help to distinguish between stretch- and resistance training-specific effects and general warm-up effects that could be induced irrespective of the intervention. Markers to quantify fatigue could furthermore provide additional information on the relationship between fatigue and the stretch-induced force deficit. However, future investigations are necessary to figure out the exact fatigue marker (central nervous system fatigue, peripheral fatigue). Therefore, interpretations remain speculative and are recommended to be addressed in future studies. 

Furthermore, results are limited to the plantar flexors. While applying reported stretching to the calf muscle under standardized conditions was enabled via the stretching device, no such device is available for other muscle groups, such as the quadriceps, hamstrings or gluteals. 

The quantification of stretching intensity is a common problem. Since Lim and Park [41] reported poor correlations between stretching pain and passive resistance of the muscle, it seems, on the one hand, problematic to quantify intensity via pain. On the other hand, this procedure is still very common in research. Nevertheless, to improve standardization and ensure using the same stretching degree, we were able to quantify the used angle of the orthosis from test to test. Even though the intensity quantification problem remained unsolved, this approach might be one step to improving the standardization of stretching study designs.

## 8. Conclusions

Both stretching and resistance training induced acute changes in flexibility and subsequent force impairments with greater deficits observed with the resistance training. Further research should explore the role of warm-up effects for acute ROM increases, while general metabolic fatigue or structural damage could possibly be responsible for strength decreases. 

## Figures and Tables

**Table 1:** Descriptive statistics and results of two-way ANOVA considering both legs for ROM of all four intervention conditions with the control leg as intraindividual control.

Parameter	Pre-Test (M ± SD) In °	Post-Test (M ± SD) In °
MVC10IL	1930.71 ± 426.32	1867.29 ± 424.77
MVC10CL	1898.36 ± 409.32	1829.93 ± 466.69
MVC20IL	1942.71 ± 461.10	1890.93 ± 451.14
MVC20CL	1865.36 ± 454.64	1898.86 ± 496.05
MVC30IL	2008.14 ± 457.48	1865.79 ± 406.70
MVC30CL	1941.50 ± 430.77	1860.86 ± 362.16
MVCSTIL	1997.36 ± 396.63	1729.86 ± 351.92
MVCSTCL	1881.86 ± 447.85	1882.14 ± 397.00
ORTH10IL	3.84 ± 2.04	5.75 ± 2.12
ORTH10CL	4.06 ± 2.06	4.61 ± 2.08
ORTH20IL	3.96 ± 1.87	5.75 ± 2.15
ORTH20CL	4.50 ± 1.80	5.00 ± 1.94
ORTH30IL	3.68 ± 2.34	5.89 ± 1.92
ORTH30CL	4.07 ± 2.39	4.68 ± 2.22
ORTHFIL	4.68 ± 2.20	4.79 ± 2.20
ORTHFCL	4.50 ± 2.43	5.00 ± 2.18
KTW10IL	13.71 ± 2.79	14.25 ± 2.96
KTW10CL	13.86 ± 2.83	14.14 ± 2.81
KTW20IL	14.07 ± 2.79	14.39 ± 3.27
KTW20CL	13.86 ± 3.26	14.21 ± 3.18
KTW30IL	14.39 ± 2.92	15.18 ± 3.21
KTW30CL	14.25 ± 2.80	14.50 ± 3.11
KTWFIL	14.11 ± 3.09	14.57 ± 3.42
KTWFCL	14.04 ± 3.05	15.32 ± 2.93

10, 20, 30 = minutes of stretching duration, CL = Control, IL = Intervention leg, KtW = knee to wall test, MVC = maximal voluntary contraction, ORTH = ROM measurement via goniometer of the orthosis.

**Table 2:** Results of the 3 × 3 ANOVA under consideration of the 3 × 3 and 2 × 2 interaction effects.

MVC 3-Way ANOVA		
*Effect*	*Significance (p-Value)*	*Effect Size (Partial eta Squared)*
Leg × Time	<0.001 *	0.62
Leg × Condition	0.19	0.34
Time × Condition	0.21	0.33
Leg × Time × Condition	0.02 *	0.57
MVC 10 min stretching 2-way ANOVA		
Time	0.07	0.24
Leg × Time	0.91	0.001
MVC 20 min stretching 2-way ANOVA		
Time	0.83	0.04
Leg × Time	0.10	0.20
MVC 30 min stretching 2-way ANOVA		
Time	0.003 *	0.52
Leg × Time	0.13	0.17
MVC RT 2-way ANOVA		
Time	0.008 *	0.43
Leg × Time	<0.001 *	0.62
ROM (ORTH) 3-way ANOVA		
*Effect*	*Significance (p-value)*	*Effect size (partial eta squared)*
Leg × Time	0.001 *	0.56
Leg × Condition	0.44	0.21
Time × Condition	0.01 *	0.62
Leg × Time × Condition	<0.001 *	0.76
ROM (ORTH) 10 min stretching 2-way ANOVA		
Time	<0.001 *	0.62
Leg × Time	0.008 *	0.43
MVC 20 min stretching 2-way ANOVA		
Time	<0.001 *	0.78
Leg × Time	0.002 *	0.55
ROM (ORTH)stretching 2-way ANOVA		
Time	<0.001 *	0.67
Leg × Time	<0.001 *	0.62
ROM (ORTH) RT 2-way ANOVA		
Time	0.06	0.26
Leg × Time	0.06	0.25
ROM (KtW) 3-way ANOVA		
*Effect*	*Significance (p-value)*	*Effect size (partial eta squared)*
Leg × Time	0.23	0.11
Leg × Condition	0.30	0.27
Time × Condition	0.75	0.10
Leg × Time × Condition	0.82	0.078
ROM (KtW) 10 min stretching 2-way ANOVA		
Time	0.02 *	0.35
Leg × Time	0.33	0.07
ROM (KtW) 20 min stretching 2-way ANOVA		
Time	0.15	0.06
Leg × Time	0.92	0.15
ROM (KtW)30 min stretching 2-way ANOVA		
Time	0.02 *	0.37
Leg × Time	0.20	0.12
ROM (KtW) RT 2-way ANOVA		
Time	<0.001 *	0.62
Leg × Time	0.59	0.02

10, 20, 30 = minutes of stretching duration, CL = Control, IL = Intervention leg, KtW = knee to wall test, MVC = maximal voluntary contraction, ORTH = ROM measurement via goniometer of the orthosis, * = *p* < 0.05

## Data Availability

The datasets used and/or analyzed during the current study areavailable from the corresponding author on reasonable request. The data are not publicly available due to containing information that could compromise the privacy of research participants.
